# miRNA in spinal muscular atrophy pathogenesis and therapy

**DOI:** 10.1111/jcmm.13450

**Published:** 2017-11-21

**Authors:** Francesca Magri, Fiammetta Vanoli, Stefania Corti

**Affiliations:** ^1^ Dino Ferrari Center Neuroscience Section Department of Pathophysiology and Transplantation (DEPT) Neurology Unit IRCCS Foundation Ca’ Granda Ospedale Maggiore Policlinico University of Milan Milan Italy; ^2^ Department of Neurological Sciences, Mental Health and Sensory Organs (NESMOS) “Sapienza” University of Rome Rome Italy

**Keywords:** spinal muscular atrophy, microRNA, biomarkers

## Abstract

Spinal muscular atrophy (SMA) is an autosomal recessive neurodegenerative disease characterized by the selective death of lower motor neurons in the brain stem and spinal cord. SMA is caused by mutations in the survival motor neuron 1 gene (*SMN1*), leading to the reduced expression of the full‐length SMN protein. microRNAs (miRNAs) are small RNAs that regulate post‐transcriptional gene expression. Recent findings have suggested an important role for miRNAs in the pathogenesis of motor neuron diseases, including SMA. Motor neuron‐specific miRNA dysregulation in SMA might be implicated in their selective vulnerability. In this study, we discuss recent findings regarding the consequences of SMN defects on miRNAs and their target mRNAs in motor neurons. Taken together, these data suggest that cell‐specific changes in miRNAs are not only involved in the SMA motor neuron phenotype but can also be used as biomarkers and therapeutic targets.

**Table:** 

● Introduction
● Rationale for studying miRNAs in SMA
● miRNA‐9 (miR‐9)
● miR‐206
● miR‐132
● miR‐183
● miR‐335‐5p
● miR‐431
● miR‐375
● miR‐2
● miR‐146
● How can miRNA alteration account for selective motor neuron death in SMA?
● miRNA as biomarkers in SMA
● miRNA as a therapeutic target in SMA
● Conclusions and perspectives
● Acknowledgements
● Conflict of interest

## Introduction

Spinal muscular atrophy (SMA) is a severe neurodegenerative disease with autosomal recessive transmission 1, 2. SMA represents the first genetic cause identified for infant mortality with an incidence of approximately one in 11,000 live births 3. The progressive degeneration of lower motor neurons located in the brain stem and spinal cord leads to muscular weakness and, at later stages, to complete paralysis 1, 2.

SMA is determined by mutations (predominantly homozygous deletions) in the survival motor neuron 1 gene (*SMN1,* MIM#600354) which encodes the full‐length form of the SMN protein 4. The *SMN1* paralogous gene, *SMN2*, predominantly encodes a truncated and unstable isoform through alternative splicing of exon 7. Only 10% of the transcript encodes a full‐length protein that can partially balance the *SMN1* absence. The number of copies of *SMN2* in the patient's genome largely determines the severity of the clinical presentation, spanning from SMA type I, the early onset and most severe form that is generally associated with two *SMN2* copies, to the less severe SMN II‐IV forms, in which patients usually harbour multiple *SMN2* copies 2. The role of the SMN protein in SMA pathogenesis and its function remain under study. This protein is ubiquitously expressed in both the cytoplasm and the nucleus, but determines a selective vulnerability prevalently in motor neurons through mechanisms that are still not completely understood 5. SMN contributes to the biogenesis of snRNPs, the major components of mRNA splicing 6. Therefore, loss of SMN protein determines aberrant splicing and widespread transcriptional changes, as demonstrated in different SMA models, to which motor neurons can be particularly susceptible 7, 8, 9. It has also been suggested that a specific essential motor neuron gene(s) is aberrantly altered, causing motor neuron loss 5. Furthermore, SMN plays an important role in neurite function, as it regulates trafficking of RNA‐binding proteins and the translation of target mRNAs at these sites 10, 11. In fact, *i*n vitro loss of SMN leads to significant defects, not only in motor neuron survival but also in neurite extension 12, 13.

In December 2016, Nusinersen (SPINRAZA™), an antisense oligonucleotide that modulates *SMN2* splicing, received approval as a therapy for all SMA types, becoming the first commercially available drug for this disease 14. Additional studies using different experimental therapies, including gene therapy and small molecules that modulate *SMN2* splicing, have shown promising results in the pre‐clinical and early clinical trial stages. In this rapidly changing environment, the definition of biomarkers to predict and follow disease progression has become increasingly crucial.

With all this in mind, a mechanism that seems to be implicated in the selective motor neuron vulnerability observed in SMA models is the alteration of the expression of motor neuron‐specific microRNAs (miRNAs). In fact, the SMN protein is likely involved in miRNA biogenesis 15, which could also explain the numerous pathways that are affected by SMN loss, as one miRNA can contemporaneously control multiple genes.

**Figure 1:**
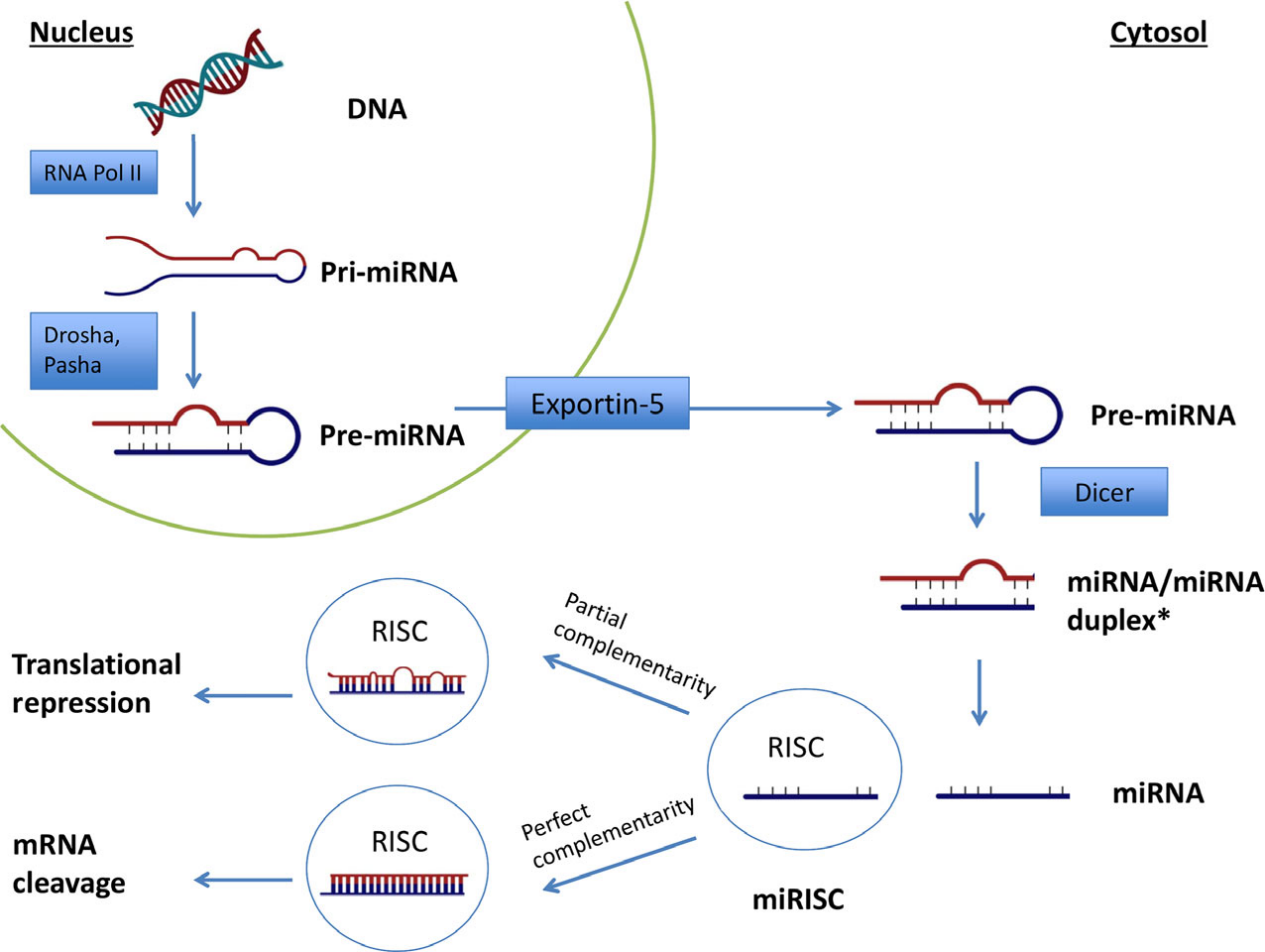
miRNA transcription and mechanism of action. The transcription of primary microRNA (pri‐miRNA) from miRNA gene is operated by RNA polymerase II (Pol II). Pri‐miRNA is then processed by *Drosha* (an *RNase III* enzyme) and its cofactor, *Pasha,* into 60‐ to 110‐nucleotide pre‐miRNA hairpins in the nucleus. The pri‐miRNA is then exported to the cytoplasm by exportin‐5 *via* nuclear pore. In the cytoplasm, pri‐miRNA is cleaved by the RNase activity of Dicer into a transient, 22‐nucleotide miRNA/miRNA* duplex intermediate. The duplex loads onto AGO2, an endonucleolytic component of the miRISC, in the RISC complex (RNA‐induced silencing complex) and separates. miRISC guides the antisense strand of the miRNA in order to complement it with the target messenger RNA (mRNA) sequence, forming a double‐stranded helix. mRNAs that bind with perfect complementarity encounter endonucleolytic cleavage. On the other hand, when there is a partial complementarity, miRNA are subject to translational repression, probably due to the formation of a bulge sequence in the middle of the helix that impedes the cleavage process.

miRNAs are small non‐coding RNAs that are approximately 22 nucleotides in length. They regulate gene expression at the post‐transcriptional level; in particular, they bind specific sequences in the 3′ UTR of target genes, inducing subsequent translational repression and/or decay of target mRNAs (Fig. 1). As mentioned above, miRNAs are able to regulate more than one target simultaneously and may function in whole networks of genes modifying protein expression 16.

## Rationale for studying miRNAs in SMA

Numerous studies have suggested the importance of miRNAs in SMA disease.

As mentioned above, SMN has a fundamental role in RNA processing. In fact, SMN binds directly to proteins relevant for miRNA generation and functionality, specifically fragile X mental retardation protein (FMRP), KH‐type splicing regulatory protein (KSRP) and fused in sarcoma/translocated in liposarcoma (FUS/TLS), which is one of the most frequent genetic causes of juvenile amyotrophic lateral sclerosis (ALS) 17, 18, 19.

In spinal motor neurons, miRNAs are essential for the development, differentiation, acquisition and maintenance of identity, axonal growth, cytoskeletal structure, synapse formation and overall activity 20, 21, 22, 23, 24, 25. The first observation suggesting the relevance of miRNA regulation in motor neurons and their relationship with SMA was made in transgenic mice harbouring a loss of *Dicer* function 26. These studies demonstrated that a lack of Dicer within motor neuron progenitors causes abnormal motor neuron development in the lateral motor column; in adult mice, it leads to progressive motor neurodegeneration resembling SMA 26, 27. These studies confirmed that miRNAs are key elements for complete motor neuron function and survival.

Because of these essential roles, it is not unexpected that dysregulation of RNA processing and miRNA expression are emerging as mechanisms involved in motor neuron diseases 28, 29. Many miRNAs are highly expressed in the central nervous system (CNS) and participate in neuronal development and differentiation. Moreover, miRNA function is indispensable for long‐term survival of spinal motor neurons *in vivo*.

Recently, it has been demonstrated that in ALS, the most common adult‐onset motor neuron disease, aberrant miRNA expression contributes to the disease pathophysiology and may represent a target for pharmacological intervention 28.

Increasing evidence also suggests that specific miRNAs can determine the selective vulnerability of motor neurons also in SMA 28, 29.

Here, we present and discuss several of these specific miRNAs involved in SMA pathogenesis and their role as potential biomarkers and therapeutic targets (Table 1).

**Table 1:** Candidate microRNAs possibly involved in SMA

	Role within CNS	Target	Expression in SMA	Dysregulation effects	References
MicroRNA‐9	Neuronal development, regulation of neuronal progenitor proliferation and the maturation and differentiation of post‐mitotic neurons; neuron dendritic outgrowth and synaptic function	*OC1, FoxP1, NEFH, Map1b, MCPIP1*	Decreased in spinal cord, increased in skeletal muscle.	Delayed neurite outgrowth *in vitro*; impairment of the radial neuronal migration in the neocortex of embryonic mouse *in vivo*	21, 22, 26, 30, 32, 33, 75
MicroRNA‐132	Neuron dendritic outgrowth and synaptic function; neovascularization	*p250GAP*	Decreased in spinal cord, increased in skeletal muscle.	Delayed neurite outgrowth *in vitro*; impairment of the radial neuronal migration in the neocortex of embryonic mouse *in vivo;* vascular defects in both spinal cord and skeletal muscle	47, 48, 49, 76
MicroRNA‐206	Myofibre formation; satellite cells differentiation; neuroprotective role in neuromuscular junction regeneration after acute nerve injury	*Pola1, BDNF, HDAC4*	Increased in both spinal cord and skeletal muscle^a^	Its up‐regulation may represent a protective response to the defective maturation of neuromuscular junction in SMA mice	39, 42, 43, 44, 45, 77
MicroRNA‐183	Protein synthesis; axonal outgrowth	*mTOR*	Increased	Inhibition of miR‐183 in a SMA mouse model increases survival and improves motor function	28, 52, 53
MicroRNA‐335‐5p	Control of differentiation or self‐renewal of mESCs	*Oct4* *pRB*	Decreased	pRB pathway inactivation antagonizes the initiation of mESC differentiation, enhancing the self‐renewal potential of mESC	55, 56, 60
MicroRNA‐431	Regulation of neuron neurite outgrowth	*Chodl*	Increased^b^	Deficits of axonal outgrowth	61
MicroRNA‐375	Development and cell survival	*PAX6, CCND2, p53*	Decreased	Higher susceptibility to DNA damage‐induced apoptosis	64, 65, 66, 67
MicroRNA‐2	Neuronal development and function; correct NMJ function	CHRM2, m2R	Decreased	Altered NMJ function	68, 69

a Normal levels in early stages of the disease, post‐natal day 5 (PND5).

b Motor neuron‐specific increase.

mESCs, mouse embryonic stem cells; Oct4**,** octamer‐binding transcription factor 4; pRB**,** protein retinoblastoma 1; Chodl, chondrolectin.

## miRNA‐9 (miR‐9)

miR‐9 is one of the most abundant miRNAs in the CNS, with essential functions as a regulator of neuronal function, generation and differentiation. In particular, miRNA‐9 has different functions during different stages of development. Overall, miR‐9 is pivotal in both spinal motor neuron differentiation and correct positioning. During early stages of development, as demonstrated in chicken embryos, miR‐9 up‐regulation influences the differentiation of neural progenitor cells into spinal motor neurons through inhibition of the transcription factor onecut1 (OC1) 21. In later stages of development, miR‐9 not only promotes differentiation but also regulates the acquisition of the identity of a subset of motor neuronal pools into the medial motor neuron column through the inhibition of the Forkhead Box P1 transcription factor (FoxP1) 22. After differentiation, miR‐9 has also been demonstrated to regulate axonal development in mammals through the inhibition of microtubule‐associated protein 1B (MAP1B) 30. Finally, later in life miR‐9 regulates motor neuron regeneration. In fact, miR‐9 expression is reduced in the early phases of nerve injury in order to initiate cell regeneration, while it is overexpressed in the later stages to reduce the expression of pro‐apoptotic genes. This pattern of expression is directly opposite to that of zinc finger CCCH‐type containing 12A (MCPIP1) – a pro‐apoptotic gene 31. All these findings contribute to our understanding that regulation of miR‐9 varies during different stages of motor neuron life, suggesting a complex dynamic phenomenon.

The first evidence suggesting the importance of miRNA dysregulation, and specifically of miR‐9, in SMA came from a study by Hornstein group that was published in 2010. In that study, conditional ablation in spinal motor neurons in rodent models of Dicer1, a protein that is fundamental for miRNA processing and maturation, altered miRNA biogenesis and led to an SMA‐like phenotype 26. Dicer1 was specifically knocked out by crossing a mouse with a Dicer1 conditional allele with an animal harbouring a Cre‐recombinase transgene under a cholinergic‐specific promoter. This allowed expression of the transgene only in post‐mitotic somatic motor neurons (at post‐natal day 7) and not during the development of these transgenic mice.

These animal models, which do not properly metabolize miRNA, showed a neuromuscular phenotype and neuropathological features that resemble those of SMA, including a loss of motor neurons, damage of the ventral horns of the spinal cord, alteration of the structure of the end plate, and atrophy and denervation of myofibres. The first modifications in animal function were detected at 2 months of age. At 4 months, both electromyography (EMG) and histopathological analysis demonstrated motor neuron cell loss. Interestingly, the neurofilament heavy subunit (NEFH), which is involved in the degeneration of motor neurons in other motor neuron diseases such as ALS, was overexpressed in the miRNA‐deficient motor neurons, supporting its key role in determining the development of the SMA phenotype. Intriguingly, this subunit is a target of miR‐9, one of the most abundant miRNAs in the CNS, and a key regulator of axonal extension and branching in neurons 30. The primary hypothesis for this observation is that the down‐regulated miR‐9 can no longer control the expression of the NEFH, resulting in its up‐regulation. These data support the relevance of miRNAs in motor neuron disease and confirm the importance of miR‐9 in regulating adult neuron survival. The pathway regulated by Dicer–miRNA is fundamental for both the CNS and peripheral nerve regeneration and axonal regrowth *in vivo* and *in vitro* 31, as demonstrated by the effects of Dicer deletion in the murine sciatic nerve 31. In the same work from the Hornstein group 26, the researchers also profiled the miRNA expression levels in an *in vitro* model represented by motor neurons derived from embryonic stem cells (ES) carrying a *SMN1* mutant allele (SMN1^mut)^. They used murine SMA ES cells from a transgenic mouse that also expressed a GFP reporter under the motor neuron‐specific promoter HB9 (Tg(Hlxb9‐GFP)1Tmj Tg(SMN2)89Ahmb Smn1tm1Msd/J); thus, they could monitor the correct differentiation of ES into motor neurons. The researchers analysed the RNA extracted from these SMN1^mut^ motor neurons, purified with FACS according to the Hb9‐GFP expression, and compared these RNA profiles with those obtained from wild‐type (WT) motor neurons. The authors demonstrated through a quantitative PCR analysis that several specific miRNAs are significantly reduced in SMN1^mut^ motor neurons. In particular, the expression levels of miR‐9 and miR‐9* (two miRNA species processed from the same hairpin) were reduced up to 15‐fold. miR‐9 is relevant to motor neuron pathology, as noted above and as demonstrated by the fact that it has neuron‐specific expression and that it is down‐regulated in *SMN1*‐deficient motor neurons. The mechanisms through which miR‐9 acts downstream of *SMN1* and through which a specific subset of SMA phenotypes is regulated by miR‐9 should be further investigated.

Wang *et al*. 32 proposed a different effect of SMN defects on miR‐9 expression. The authors down‐regulated SMN expression using gene‐specific inducible short hairpin RNA interference (shRNAi) in the motor neuron‐like cell line NSC34 and in human skin fibroblast cells. In these experiments, they observed that miR‐9 levels showed a time‐dependent increase when cellular SMN protein levels were diminished. In a complementary manner, cellular miR‐9 levels decreased over time after SMN overexpression in HeLa cells. They also analysed the spinal cords of SMA mice and human primary skin fibroblasts isolated from patients with different degrees of SMA disease severity. In these cellular models, miR‐9 levels were increased proportionally to the reduction in SMN. These data suggest a possible role for miR‐9 expression in certain cells as a prognostic marker, as its expression appears to be regulated by the SMN protein and directly correlates with the severity of SMA phenotype.

Catapano *et al*. 33, in line with the previous work of Haramati *et al*. 26, demonstrated a significant down‐regulation of miR‐9 in the spinal cord of SMA I mice compared with controls. By contrast, they observed a greater than threefold increase in miR‐9 in skeletal muscle. Systemic treatment with morpholino oligonucleotides, which modulated *SMN2* splicing to allow production of a large quantity of full‐length protein, normalized miR‐9 levels in skeletal muscle but not in the spinal cord, further supporting miR‐9's potential utility as a biomarker. Higher levels of miR‐9 were also detected in the serum of SMA mice, and were reduced by morpholino treatment that up‐regulated SMN; these miRNA level changes occurred much earlier than the changes in level in the spinal cord and skeletal muscle. miR‐9 levels were also shown to be increased in the serum of SMA type II and, to a lesser extent, in type III patients, who showed levels that were intermediate between SMA type II patients and healthy controls.

Overall, the studies concerning miRNA‐9 expression in SMA suggest that miR‐9 levels are significantly altered by the reduction of the SMN full‐length protein. miR‐9 expression can be down‐ or up‐regulated depending on the SMA cells or tissues; it is likely down‐regulated in the CNS/motor neurons and up‐regulated in other non‐neuronal cells and in the blood. However, knockdown of SMN in a motor neuronal cell line resulted in miR‐9 up‐regulation 28, and miR‐9 expression is increased in the ALS SOD1G93A mouse spinal cord 34. These observations highlight the difficulties in generalizing a conclusive profile of miR‐9 expression in diseased motor neurons. However, in a defined SMA pathological model, the miR‐9 pattern of expression is restored to the wild‐type ‘normality’ by the up‐regulation of SMN expression, indicating its potential utility as a biomarker.

## miR‐206

Muscle involvement in SMA patients is related not only to skeletal muscle denervation secondary to spinal motor neuron defects but also to disruption of the myogenic program determined by SMN deficiency 35, 36. miR‐206 is a miRNA that is primarily expressed in muscle. It regulates the creation of myofibres and differentiation of satellite cells 37. Although miR‐206 has been reported mainly to be a skeletal muscle‐specific miRNA, its expression has also been detected in spinal motor neurons 38 where it has a role in the process of regeneration of neuromuscular synapses 39, 40 after nerve injury.

miR‐206 regulates the processes of motor axonal growth and muscle connection after acute nerve injury by influencing the expression of fibroblast growth factors (FGFs) and brain‐derived neurotrophic factor (BDNF), which are key elements of motor neuron regeneration.

After acute nerve injury in mice, miR‐206 is up‐regulated and promotes distal axon regrowth and muscle reinnervation through inhibition of the mRNA of histone deacetylase 4 (*HDAC4*), with consequent increase of fibroblast growth factor binding protein (FGFBP1) 39. This is secreted into the extracellular space, where it enhances FGF‐7 action to elicit distal axon regrowth and muscle reinnervation 41. Moreover, miR‐206 may play a role as a negative regulator of BDNF in skeletal muscle 42.

In a pathological context, such as in ALS, miR‐206 has a neuroprotective role and is involved in the regeneration of neuromuscular junctions (NMJs) 39, 40. In fact, miR‐206 silencing in an ALS mouse model accelerated disease progression 39. In mouse models of both muscular dystrophy and ALS, the miR‐206 serum level is increased in a possible compensatory manner 43. A significantly increased expression of miR‐206 has been described in both the skeletal muscle and serum samples of ALS patients 44, supporting its relevance as a possible biomarker for this disease, even though further studies in larger cohorts remain necessary. While skeletal muscle expression of miR‐206 can exert a positive role, the serum expression may be more likely related to the rate of clinical worsening 44.

In SMA models, interestingly, the increase in miR‐206 levels in muscle delays neurodegeneration, despite not being able to completely rescue the integrity of motor neurons 45.

The authors of this study used the SMNΔ7 mouse model to analyse the correlation between miRNA‐206 levels and NMJ architecture. They demonstrated that the quadriceps, a muscle affected earlier in this SMA model, shows a morphological pattern characterized by an increase in atrophic fibres, a fibre type disproportion with a reduction of type II fibres, and an accumulation of neurofilaments in the pre‐synaptic terminal of the NMJs. Furthermore, molecular investigation showed that miRNA‐206 also has an effect in regulating the HDAC4‐FGFBP1 pathway. In fact, as mentioned above for wild‐type rodents, miRNA‐206 up‐regulation determines the HDAC4 protein reduction and consequent increase in FGFBP1 mRNA. This pathway has a neuroprotective effect and increases re‐innervation of muscle endplates. Overall, miR‐206 may play a significant protective role in NMJs and muscle and could represent a possible as yet unexplored therapeutic target for SMA.

Treatment with oligonucleotides to increase SMN does not significantly modify miRNA‐206 levels in SMA I mice, either in the spinal cord or in skeletal muscle, but does normalize its serum levels 33.

It is intriguing that miRNA‐206 is commonly dysregulated in both SMA and ALS. In both of these disorders, miR‐206 has been shown to be able to partially delay the neurodegeneration process. miRNA 206 is capable of modulating the expression of several target genes that could represent several common molecular pathways underlying both ALS and SMA, thus representing potential therapeutic targets for these diseases.

## miR‐132

Another miRNA highly represented in the CNS and with a putative role in SMA disease is miR‐132. Its role is mainly in the modulation of neuronal dendritic outgrowth and synaptic function 46, 47. miR‐132 can delay neurite outgrowth *in vitro* and might interfere with radial neuronal migration in the embryonic mouse neocortex *in vivo* 48. miR‐132 can be involved in the neovascularization process 49, in particular in the vascular defects that have been observed in the skeletal muscles and spinal cords of SMA models 50, 51. Catapano *et al*. 33 showed a reduction of miR‐132 levels in the spinal cord of SMA I mice, which was reversible with morpholino therapy. miR‐132 levels in muscle were normal, while serum levels were elevated; these normalized after oligonucleotide therapy. miR‐132 expression is also up‐regulated in type II SMA patient sera, whereas the level in SMA III patient sera was intermediate with respect to SMA II and healthy control sera. This pattern of tissue gene expression resembles that of miR‐9.

## miR‐183

Studies of miRNA expression demonstrate a different miRNA profile in primary SMA cortical neurons with respect to control tissues 52. Both animal and cellular models of SMA show an increased miR‐183 level in fibroblasts, motor neurons and cortical neurons 52.

Specifically, researchers have found high levels of miR‐183 in neurites of SMN‐deficient neurons and demonstrated how miR‐183 up‐regulation is able to suppress axonal growth 52. Importantly, both the altered expression and the altered distribution seen with increasing concentrations in neurites might influence this process.

Moreover, it has been demonstrated that the translation of mechanistic target of rapamycin (mTOR) is regulated by miR‐183 through its direct binding to the 3′ UTR 50. mTOR is a serine/threonine kinase that forms two different complexes: mTOR complex 1 (mTORC1) and mTOR complex 2 (mTORC2). These complexes have been demonstrated to regulate axon regeneration 53, cellular cytoskeleton stability and mitochondrial biogenesis/degradation 54.

In SMN‐deficient neurons, the local axonal translation of mTOR appears to be reduced. *In vivo* experiments have demonstrated the possibility of restoring it by inhibiting miR‐183. This inhibition also has functional consequences, as demonstrated by the fact that inhibition of miR‐183 in the spinal cord in a SMA mouse model resulted in an increase in median survival as well as motor function improvement 52. These data imply that miR‐183, the mTOR pathway and the tight regulation of the miR‐183/mTOR balance may represent crucial molecular mechanisms involved in SMA pathology. The role of basal miR‐183 expression in the regulation of motor neuron function and survival remains to be determined. However, several studies have demonstrated that even if increased expression of miR‐183 has a negative effect on motor neurons, basal expression levels and transient increases in miR‐183 might be positive for motor neuron function. These observations highlight the need for further study regarding the use of miR‐138 as a therapeutic target, as this will require precise modulation of its levels.

## miR‐335‐5p

Recently, it has been demonstrated that miR‐335‐5p, which is known to be able to control differentiation and self‐renewal of mouse ES 55, is also involved in SMA pathology. In fact, SMN∆7 SMA and wild‐type (WT) neural progenitor cells derived from E13.5 mice spinal cords have different expression levels of miR‐335‐5p. Furthermore, it appears that miR‐335‐5p may also be associated with the increased proliferation found in SMA cells 56.

Luchetti and his group 56 described for the first time the characteristics of neural stem cells (NCSs) derived from the spinal cords of a severe *SMNΔ7* SMA mouse model, comparing them to WT‐derived NSCs. While several characteristics remained the same, such as the ability to form neurospheres and the expression of specific regional and self‐renewal markers, *SMNΔ7* NSCs had an altered cell cycle distribution as well as an increased proliferation rate compared to WT‐NSCs. These differences in proliferation may be observed because in *SMNΔ7* NSCs, the expression of certain miRNAs, such as miR‐100‐5p and miR‐335‐5p, is reduced, further highlighting the potential role of miRNAs in SMA pathology 56.

In the past decade, there has been growing interest in utilizing induced pluripotent stem cells (iPSCs) as an *in vitro* model for genetic disorders, because they offer the important possibility of studying genetic diseases directly in human cells that are not accessible in live patients, such as motor neurons.

*In vitro* models of SMA using human iPSCs have already been described 57. Specifically, we and other groups have demonstrated that motor neurons derived from iPSCs from type I SMA patients show low levels of the SMN protein, undergoing selective death over time 8, 58, 59.

In a recent study, Murdocca *et al*. 60 analysed the molecular and phenotypic features of SMA human iPSCs, specifically the gene expression and cell cycle proliferation of SMA and WT iPSCs in the process of differentiating into early motor neurons. Their study confirmed a reduced level of miR‐335‐5p in SMA cells, which could be associated with a low expression of early motor neuron markers 60.

Using quantitative reverse transcription PCR (qRT‐PCR), it was possible to demonstrate that while the expression of miR‐335‐5p is almost equivalent between SMA and WT iPSCs, it begins to significantly differ at both 14 and 22 days of differentiation. After 14 days, miR‐335‐5p appears to be down‐regulated more than twofold in SMA versus WT cells, and after 22 days, the difference is even more pronounced. This evidence suggests that miR‐335‐5p may be involved in the process of differentiation of iPSCs towards early motor neurons 60.

These new results regarding the correlation between down‐regulation of miR‐335‐5p in differentiating cells and SMA are important, as they suggest an involvement of this specific miRNA in SMA pathology specifically in cells that are differentiating towards motor neurons.

As previously reported, at the iPSC stage there is no difference in miR‐335‐5p expression, while the difference becomes more significant after 2 and 3 weeks of differentiation. This evidence deserves further in‐depth analysis in order to better understand how miR‐335‐5p down‐regulation is involved in SMA precursor differentiation and, most importantly, its possible correlation with SMA phenotype.

## miR‐431

The Sahin group 61 demonstrated that SMN knockdown alters miRNA expression with a pattern that is specific for each type of cell. The aim of their study was to define the consequences of SMN loss in miRNAs and their target mRNAs in spinal motor neurons. RNAseq was used to analyse both miRNA and mRNA expression in primary spinal cord motor neuron cultures after acute SMN loss. This *in vitro* model was created using wild‐type embryonic rat spinal motor neurons infected with lentiviral shRNA specific for down‐regulating SMN (shSMN). Both Western blot and qRT‐PCR analyses were used to evaluate SMN knockdown. After 6 days, these methods demonstrated an 80% and 75% reduction, respectively, in SMN protein levels 61.

Through the integration of miRNA/mRNA profiles, it was possible to detect a number of dysregulated miRNAs (*n* = 17) with increase in several of their putative mRNA targets. The expression of miR‐431 was very high, while a number of its putative target mRNAs were considerably down‐regulated in motor neurons with reduced SMN levels. It has been previously described that miR‐431 is expressed in the brain as well as in the spinal cord during embryonic development 62.

**Figure 2:**
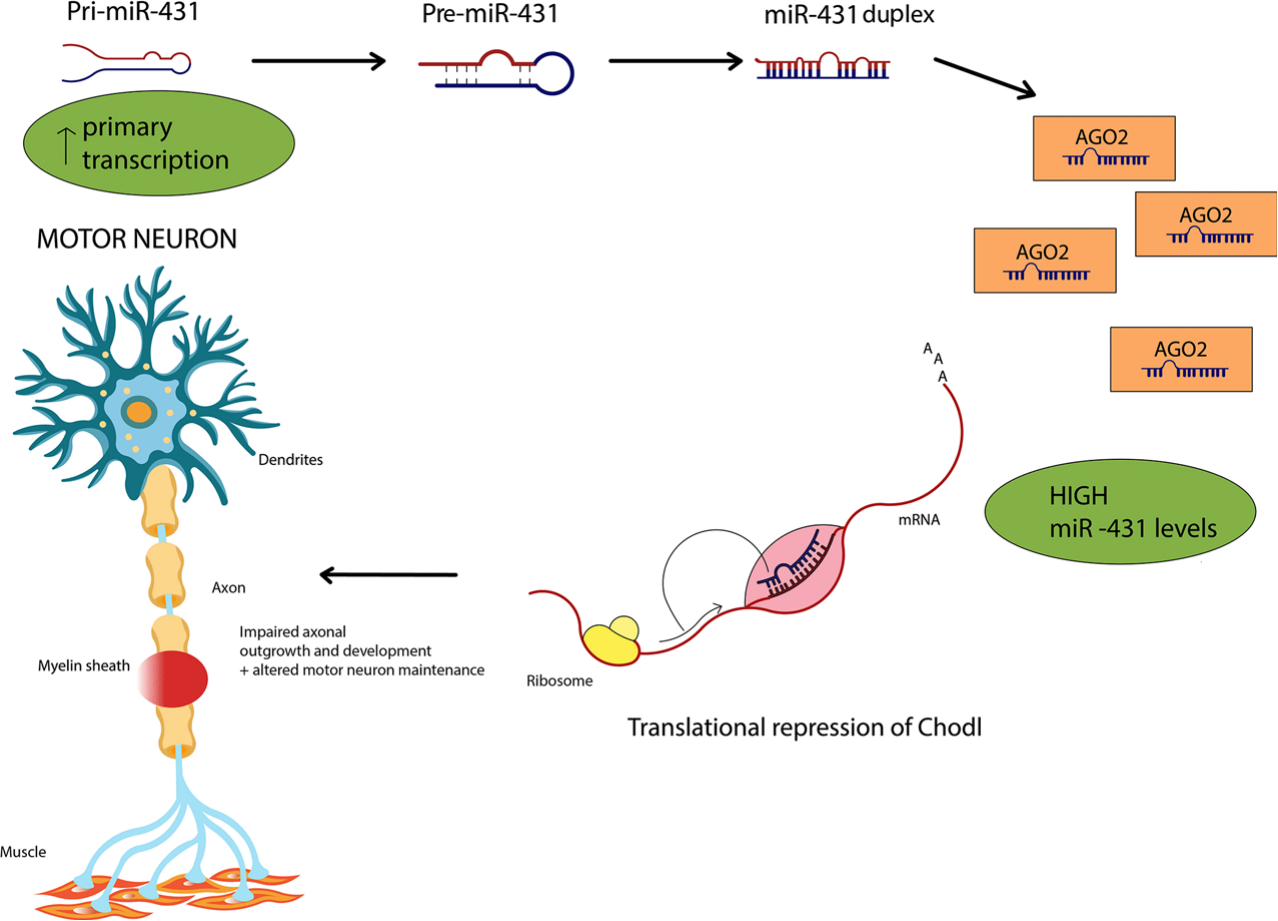
Increased primary transcription, as testified by high levels of pri‐miR‐431, leads to high miR‐431 levels. There is a set of miR‐431 genes that are down‐regulated in SMN knockdown, among which chondrolectin (Chodl), a C‐type lectin, is a key regulator of motor neuron outgrowth. miR‐431 binds to the *Chodl* 3′UTR site inducing its translational repression and decay. Low Chodl levels translate into impaired axonal development and outgrowth, a core feature of SMA. Interestingly, in SMN knockdown miR‐431 levels are normal in fibroblasts and cortical neurons, making its increased expression a specific feature of motor neurons.

Interestingly, by studying iPSC‐derived motor neurons of type I SMA patients, it was possible to detect a marked increase in miR‐431. This change appears to be specific to motor neurons, because it was not present in fibroblasts derived from type I SMA patients. By measuring the primary miR‐431 transcript level (pri‐miR‐431), which was also increased, it was possible to establish that the mechanisms underlying the miR‐431 increase may include its augmented primary transcription. Moreover, Wertz *et al*. discovered that miR‐431 was also involved in the regulation of neuronal neurite outgrowth (Fig. 2). Therefore, by inhibiting the overexpression of miR‐431 in SMN‐deficient motor neurons, it is possible to rescue the motor neuron neurite length phenotype. The authors also identified chondrolectin (Chodl), a type 1 transmembrane protein and member of the c‐type lectin domain‐containing family 61, as a direct target of miR‐431. Chodl is fundamentally important because it regulates the outgrowth of motor neurons axons. This observation suggests that the lack of axonal outgrowth in SMA motor neurons may be due to a down‐regulation of Chodl caused by an increase of miR‐431 61.

## miR‐375

miR‐375 has two important functions: it contributes to the promotion of the acquisition of a motor neuron phenotype and protects neurons from apoptosis after DNA damage.

During development, miR‐124 promotes neurogenesis through RE1‐silencing transcription factor (REST) silencing 63. REST is a transcription factor (TF) that plays a key role in regulating the balance between self‐renewal and neuronal differentiation, suppressing neuronal genes in non‐neuronal tissues. Moreover, it plays a crucial role in various stages of neuronal development, such as the differentiation of embryonic stem cells (ESCs) into neural stem cells (NCSs), and further differentiation of NCS into neurons. Bhinge *et al*. investigated which miRNAs were regulated by REST using *Rest*‐null mouse ESC, in which normally suppressed miRNAs should be highly activated 62. Among various miRNAs that were differentially expressed in response to Rest knockout, Bhinge's group focused on miR‐375, which was not only up‐regulated after Rest knockout, characterizing it as one of Rest's direct targets, but was also highly expressed during neuronal differentiation of wild‐type ESC, thus likely being implicated in neurogenesis 62. Using an *in vitro* model of spinal motor neuron development, Bhinge *et al*. discovered that miR‐375 is highly activated during spinal motor neurogenesis and that its expression is specific to MNs, indicating an essential role for miR‐375 in motor neuron development.

miR‐375 promotes motor neuron differentiation through silencing PAX6 and cyclin D2 (CCND2) transcript expression, both of which are direct targets of miR‐375. This silencing enhances motor neuron differentiation 64; PAX6 is also important for the generation of neural progenitor cells and for the correct balance between self‐renewal, patterning and differentiation in the developing spinal cord.

The timing of REST silencing is fundamental. In fact, in order to allow the formation of post‐mitotic spinal motor neurons, miR‐124 must silence REST activity and miR‐375 must suppress PAX6 at specific time‐points 63, 64.

The second major function of miR‐375, the protection from apoptosis, is mediated by reduction of tumour suppressor p53 protein levels 64. According to its pro‐apoptotic function, p53 levels remain low in mature neurons. After DNA damage, developing motor neurons show an increase in p53 expression, which leads to programmed cell death 65. It has been demonstrated that overexpression of miR‐375 can prevent cell death after DNA damage 64.

p53‐mediated neuronal apoptosis has been described in various neurodegenerative disorders, including ALS and SMA 66, 67.

Bhinge *et al*. observed that in SMA motor neurons, p53 levels were remarkably high while miR‐375 levels were extremely low 64. This observation is also consistent with the fact that SMA MNs are much more susceptible to DNA damage compared to healthy MNs. Exogenous expression of miR‐375 was able to rescue SMA motor neurons from cisplatin‐induced apoptosis, strengthening the hypothesis that miR‐375 exerts a protective effect by inhibiting p53 and has a general protective role in spinal MNs 65. The identification of these new regulatory mechanisms involved in SMA pathogenesis may lead to new potential therapeutic approaches for SMA.

## miR‐2

The possible role of miR‐2 in SMA pathogenesis has recently been demonstrated.

In mice, miR‐2 acts as a negative regulator of m2R (a muscarinic acetylcholine receptor important for neurons) and as an inhibitor of cholinergic MN synaptic release 68. miR‐2 is a specific target of Gemin3, which is a protein that interacts with SMN and participates in microRNA processing. In particular, Gemin3 is an RNA helicase that correlates both with SMN and RNA‐induced silencing complex components and may also play a role in spliceosome assembly, mRNA transport and miRNA function in general 68.

Recently, Patrick O'Hearn *et al*., using a *C. elegans* model, reported that the reduction of SMN levels can cause neuromuscular junction (NMJ) dysfunction in motor neurons and suggested that dysregulation of neurotransmission may represent another pathological mechanism in SMA 69.

According to their model, loss of the *C. elegans* SMN orthologue SMN‐1 causes mir‐2 and consequently GAR‐2 (*C. elegans* m2R orthologue), miR‐2 dysregulation and NMJ defects. Increasing in C. elega*ns* the Gemin3 orthologue MEL‐46 modifies miR‐2 expression and ameliorates NMJ defects.

GAR‐2 and m2R are functionally conserved, as activation of these pre‐synaptic receptors by acetylcholine in different species results in hyperpolarization and decreased NMJ acetylcholine release across species, and several treatments that block m2R are currently available to treat other conditions.

In a SMA mouse model, m2R levels showed an increase of 50% compared to controls, and pharmacological inhibition of m2R by methoctramine was able to rescue MN phenotype and to overcome MN axon outgrowth defects.

Collectively, these results suggest that m2R up‐regulation in SMA at early stages may contribute to MN dysfunction, resulting in overactive neurotransmission and that therapies that target miR‐2 expression can modify disease evolution.

## miR‐146

The precise mechanisms that lead to selective motor neuron loss in SMA are currently under investigation, but it is well known that extrinsic influences, such as those arising from astrocytes, may also contribute to SMA pathogenesis. Among the different mechanisms suggested, altered expression of miRNAs in astrocytes has recently been highlighted. Sison *et al*. specifically investigated miRNA expression and secretory profiles in SMA astrocyte models, and they demonstrated that miR‐146a was the only miRNA that was up‐regulated 70. This miRNA is not overexpressed in motor neurons and appears to have a role in the regulation of the immune system response to interleukins, TNF‐α and glial activation 71. miR‐146 exerts its action by down‐regulating glial‐derived neurotrophic factor (GDNF), which is a potent survival factor for a number of neuronal subtypes 72, and by acting on NOTCH signalling. miR‐146 levels are influenced both directly and indirectly by SMN1 levels. SMN re‐expression decreases miR‐146a levels nearly to control levels, and the NFκB pathway is instead an inducer of miR‐146a. The pathogenic role of this miRNA is demonstrated by the fact that treatment of iPSC‐derived motor neurons *in vitro* with synthetic miR‐146a molecules can mimic SMA pathogenesis and induce motor neuron loss. Moreover, miR‐146a inhibition rescues the SMA phenotype 70 Together, these data indicate that altered astrocyte production of miR‐146a may be a contributing factor in astrocyte‐mediated SMA pathology and that miR‐146a regulation, such as through regulation of NFκB signalling, could represent an additional therapeutic target for the treatment of SMA.

## How can miRNA alteration account for selective motor neuron death in SMA?

The critical role of the SMN protein in miRNA biogenesis and processing confirms the importance of miRNAs in SMA pathogenesis Overall, miRNAs have multiple functions and appear to be fundamental for neuronal survival. However, the expression of miRNA is highly variable in different neuronal cells and in different stages of cell development. In fact, each neuronal subtype is characterized by a specific miRNA profile that changes over the course of the development, maintenance and regeneration phases. Their highly specialized morphology and function make motor neurons strictly dependent on precise miRNA functioning and regulation.

One of the main questions in the miRNA/SMA field is how to interpret miRNA specificity for diseases, as miRNAs are involved in several functions related to neuronal involvement. The link between miRNA disruption and selective motor neuron death likely resides in their targets.

For example, miR‐9 alteration leads to reduced down‐regulation of heavy neurofilaments, a pathogenic process that specifically involves motor neurons. In fact, neurofilament accumulation has been supported by several studies as one of the more likely causes of selective motor neuron degeneration. The neurons that are most prominently affected by the accumulation of neurofilaments are the largest neurons with the longest axons, which normally contain abundant neurofilaments. Indeed, cytoskeletal abnormalities may augment the susceptibility of neurons to other insults, for example excitotoxicity, so that multiple elements culminate in the disease 31.

The miR‐183/mTOR pathway regulates local axonal translation and axonal growth, which are fundamental in motor neurons. Interestingly, miR‐183 partially rescues the neuronal phenotype induced by SMN loss, and can be an additional target besides the modulation of the mTOR pathway.

Also miRNAs which are specifically altered in astrocytes, such as mi‐146, can influence motor neuron development due to their high dependence on extrinsic influences.

Current knowledge confirms the importance of changes in miRNA expression, especially in early disease stages in the miRNA–mRNA regulatory network in the pathogenesis of SMA. Although several pathways, targets and intersections have now been determined, a more detailed understanding of the transcriptome networks in both healthy and diseased motor neurons is fundamental to establishing further understanding of the pathogenesis and mechanisms that lead to selective motor neuron susceptibility.

## miRNA as biomarkers in SMA

Despite significant progress in the understanding of SMA pathogenesis, definition of the biomarkers for this disease is still lacking. With promising therapies progressing through clinical development, there is an urgent need for the definition of molecular biomarkers that could be fundamental to performing patient classification, following disease evolution and better defining the efficacy of therapeutic approaches using minimally invasive procedures.

Overall, miRNAs hold an interesting diagnostic and prognostic potential as non‐invasive biomarkers for SMA. In fact, miRNAs are relatively stable in accessible biofluids, such as blood and CSF, and can be detected with accessible and feasible laboratory methods. Moreover, multiple samples can be captured in order to follow disease evolution over time.

Among the miRNAs highlighted in this review, miR‐9, miR‐132, miR‐206, miR‐183 and miR‐375 could represent possible biomarkers for SMA, while miR‐183 and miR‐375 may play additional roles in contributing to the specificity of SMA determination, based on the data presented above in each miRNA subchapter. The major critical challenges remain the possible inconsistencies in the results due to human subject variability and technical issues related to the relative fragility of miRNAs. In general, one of the critical aspects is the specificity of this kind of biomarker.

The recruitment of large cohorts of patients could increase the power of biomarker studies and promote the advancement of the field.

## miRNA as a therapeutic target in SMA

Over the last several years, given their specific ability as broad transcriptomic regulators, miRNAs have been proposed as promising therapeutic targets for neurodegenerative diseases, including motor neuron disorders. Schematically, there are two possible approaches to modulating miRNAs: (*i*) overexpressing them with miRNA mimics or (*ii*) down‐regulating them through antisense oligonucleotides (anti‐miRNAs) 73.

However, there are many issues that must be overcome and that make it difficult to use miRNA for therapeutic purposes. One of these challenges is the method of delivery: these molecules can be delivered either naked to the target tissues, at the price of being quickly degraded or having limited cell penetration, with some modifications (peptide), or with the use of delivery tools such as nanocapsules 73. The most effective route of delivery must also be determined: intrathecal injection allows higher concentrations and more selective action but is more invasive; therefore, systemic administration should also be considered. Furthermore, it must be considered that miRNAs often act through multiple pathways and that there is the risk that they may influence transcription in off‐target tissues, leading to the possibility of significant side effects and adverse events. Finally, the timing of miRNA regulation according to their cellular developmental status must also be considered, as miRNA levels should be modulated at the correct stage of development.

Among the miRNAs presented in this review, miR‐9, miR‐206, miR‐183 and miR‐375 could potentially be explored as therapeutic targets for SMA.

miR‐9 represents one of the most interesting targets because it is the one with the most relevance to motor neuron function. The vast majority of experimental evidence suggests that miR‐9 is generally down‐regulated in SMA spinal cords. Thus, one possible therapeutic strategy could be its up‐regulation *via* gene/molecular therapy. However, miR‐9 is a key player in many processes within many cells. Its expression is tightly regulated and can vary significantly between each phase of motor neuron regeneration, being first up‐regulated and later down‐regulated. Thus, artificially mimicking these complex variations with pharmacological or gene therapy without inducing significant off‐target effects may be difficult. Indeed, the findings of miR‐9 down‐regulation are not consistent in all data sets in the literature, and conflicting results have been observed depending on the SMA model/cell type considered. In any case, the testing of miR‐9 as a therapeutic target warrants further investigation in pre‐clinical SMA models.

miR‐206 may play a significant protective role in NMJs and muscles and represents a potential unexplored therapeutic target for SMA.

Regarding miR‐183, its expression in the spinal cord of an SMA mouse prolonged survival and improves motor function of Smn‐mutant mice 52; therefore, miR‐183 might also represent an interesting target.

Finally, miR‐375 can confer motor neuron protection by shielding motor neurons, *via* inhibition of p53, from increased susceptibility to DNA damage‐induced apoptosis 65.

miRNA therapeutic strategies can also be used to directly up‐regulate SMN levels.

It has been recently demonstrated in *zebrafish* that the expression of anti‐*smn1* miRNAs in motor neurons reproduced most of the hallmarks observed previously in the ubiquitous knockdown model. In addition, *smn1* knockdown in *zebrafish* motor neurons is sufficient to induce late‐onset motor neuron degeneration 74. Based on this observation, it is possible to determine a strategy to block anti‐SMN1 miRNA with anti‐miRNAs, with the goal of up‐regulating SMN levels.

The demonstration of the expression of anti‐*SMN1* or *SMN2* miRNAs in mammalian motor neurons is the first step to further developing this idea.

## Conclusions and perspectives

miRNA dysregulation likely has an important role in SMA pathogenesis and in determining the selective vulnerability of motor neurons. In particular, miRNA activity seems to be fundamental for the survival and function of post‐mitotic spinal motor neurons. miRNA homeostasis disruption in murine spinal motor neurons contributes to the appearance of typical hallmarks of SMA. Different studies have shown altered expression of specific miRNAs in SMA, and several of these experiments have highlighted the same miRNAs in different SMA models. The miRNAs related to neuronal function are those most frequently identified as aberrantly expressed, even if the exact molecular mechanisms in which they are involved are largely undefined. Further investigations of this perspective will allow increased understanding of how miRNAs intervene in SMA pathogenic mechanisms. The expression profile of each miRNA can vary in different tissues, such as muscle, serum and spinal cord, and can even reflect the severity of SMA, being more altered in SMA I than SMA II and SMA III.

Therefore, miRNAs can be used not only as biomarkers of pathology but also as markers of disease severity and progression, as well as of response to effective experimental therapies in future clinical trials. Furthermore, the therapeutic potential of miRNAs must be thoroughly explored. Given that *in vitro* and *in vivo* experiments have demonstrated the neuroprotective potential of certain miRNAs, this would be an important topic to be assessed in subsequent studies for implementing novel SMA therapies. For instance, miRNA can be modified by RNA therapy, including antisense oligonucleotides, miRNA mimics and antagomirs, and could represent a promising potential therapeutic target for SMA in association with SMN‐targeted therapies.

## Funding source

The authors have no relevant affiliations or financial involvement with any organization or entity with a financial interest in or financial conflict with the subject matter or materials discussed in this manuscript.

## Conflicts of interest

The authors confirm that there are no conflict of interests.
